# Supplementary material for: Topoisomerase I is an evolutionarily conserved key regulator for satellite DNA transcription
Source: Nat Commun. 2024 Jun 17;15:5151. doi: 10.1038/s41467-024-49567-5 (PMC11183047; doi:10.1038/s41467-024-49567-5)
Supplement: Supplementary file 1 — Supplementary Information [file 41467_2024_49567_MOESM1_ESM.pdf]

SUPPLEMENTARY INFORMATION

Topoisomerase I is an Evolutionarily Conserved Key Regulator for Satellite DNA Transcription

Zhen Teng[1,5], Lu Yang[1,5], Qian Zhang[1,5], Yujue Chen[1], Xianfeng Wang[1], Yiran Zheng[1], Aiguo Tian[1,2,3], Di Tian[4], Zhen Lin[2,4], Wu-Min Deng[1,2], and Hong Liu[1,2,3] *

* Correspondence, all inquiries should be addressed to: hliu22@tulane.edu

This file includes:

Supplementary Figure legends 1 to 11

Supplementary Figures 1 to 11

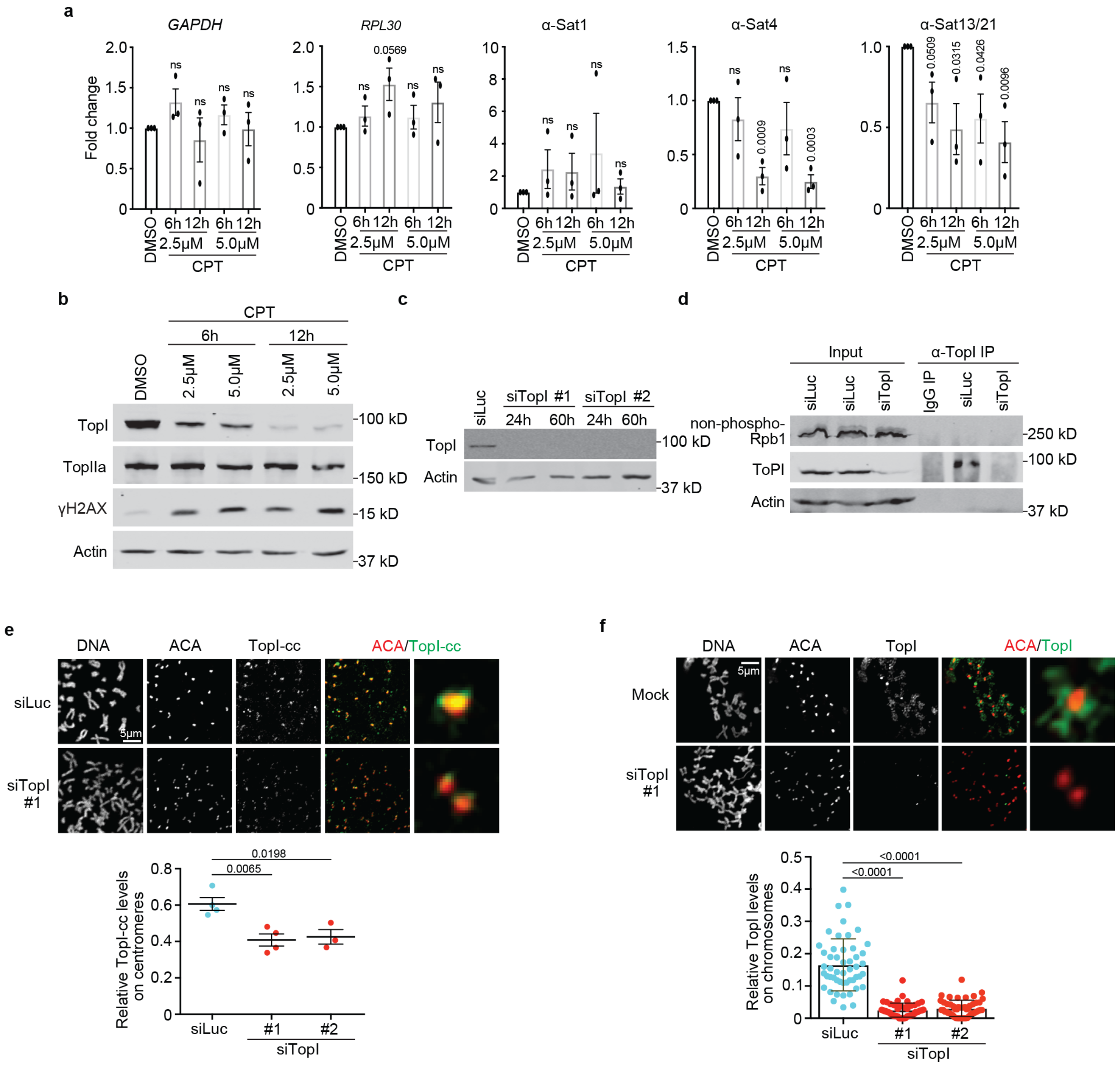

Supplementary Fig. 1. TopI promotes α-satellite transcription

a. TopI inhibition decreases centromeric transcription. HeLa cells were treated with CPT (2.5 μM) at distinct doses for 6 or 12 hrs and RNAs were extracted for real-time PCR analysis. Biological replicates n=3. Data are presented as mean values with +/- SEM. Two-sided Student's T-test. **b.** Lysates of HeLa cells in (**a**) were subjected to Western blotting analysis. **c. Knockdown efficiency of TopI siRNA.** Lysates of HeLa cells transfected with luciferase or TopI siRNAs (#1 or #2) at different timepoints were subjected to Western blotting analysis. **d. TopI does not physically interact with non-phosphorylated RNAP II**. Lysates of HeLa cells transfected with luciferase siRNA or TopI siRNA#1 were subjected to coimmunoprecipitation

with antibody against non-phosphorylated RNAP II (8WG16). Results for coimmunoprecipitation with antibodies against total RNAP II (4H8) and phosphorylated RNAP II (Ser2) (H5) were recorded in Fig. 2d. **e** and **f. Validation of TopI and TopI-cc fluorescence signals.** HeLa cells were transfected with luciferase siRNA or TopI siRNA#1. After a brief nocodazole (1.5 hr, 5 μM) treatment, mitotic cells were collected for immunostaining. Quantifications of TopI-cc levels (TopI-cc/ACA, **e**) and TopI levels (TopI/DNA, **f**) are shown in the bottom panels. Biological replicates n=4 (siLuc and #1, **e**) and n=3 (#2, **e**). For (**f**), similar results were obtained for two biological replications and quantification was performed based on a single experiment. One dot represents one chromosome. n=50 for each condition. The data here are presented as mean values +/- SEM in (**e**) and mean values +/- SD in (**f**). Two-sided Student's T-test for (**e**, **f**). For Western blots in (**b**, **c**, and **d**), similar results were obtained in at least two biological replicates. The statistical analysis in (**f**) was carried out based on a single experiment. Details of quantification and technical details in all the supplemental figures are recorded in the section of Methods. ns, not significant (P>0.1). Numeric values for P<0.1 are shown. The source data are provided in the Source Data file.

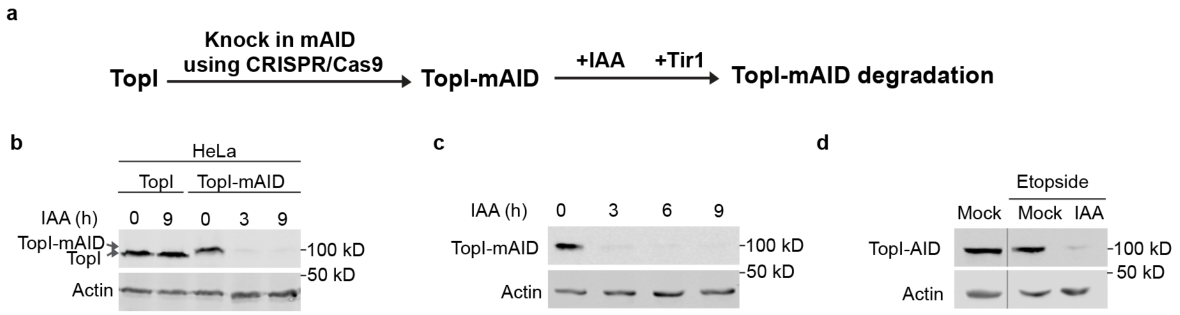

Supplementary Fig. 2. Construction of TopI-mAID cell lines

a. Schematic for construction of TopI-mAID**. b.** Parent HeLa cells and Top-mAID HeLa cells were treated with IAA (1 μM) at the indicated times. Cell lysates were analyzed by Western blotting. **c.** Top-mAID HeLa cells were treated IAA at the indicated times. Cells were collected for real-time PCR analysis (recorded in Fig. 1d) and Western blotting analysis. **d.** Top-mAID HeLa cells were treated IAA (1 μM) and etoposide for 9 hrs. Cells were collected real-time PCR analysis (recorded in Fig. 3d) and Western blotting analysis. These blots were spliced from the same gel (Source Data file). For Western blots in (**b**, **c**, and **d**), similar results were obtained in at least two biological replicates. The source data are provided in the Source Data file.

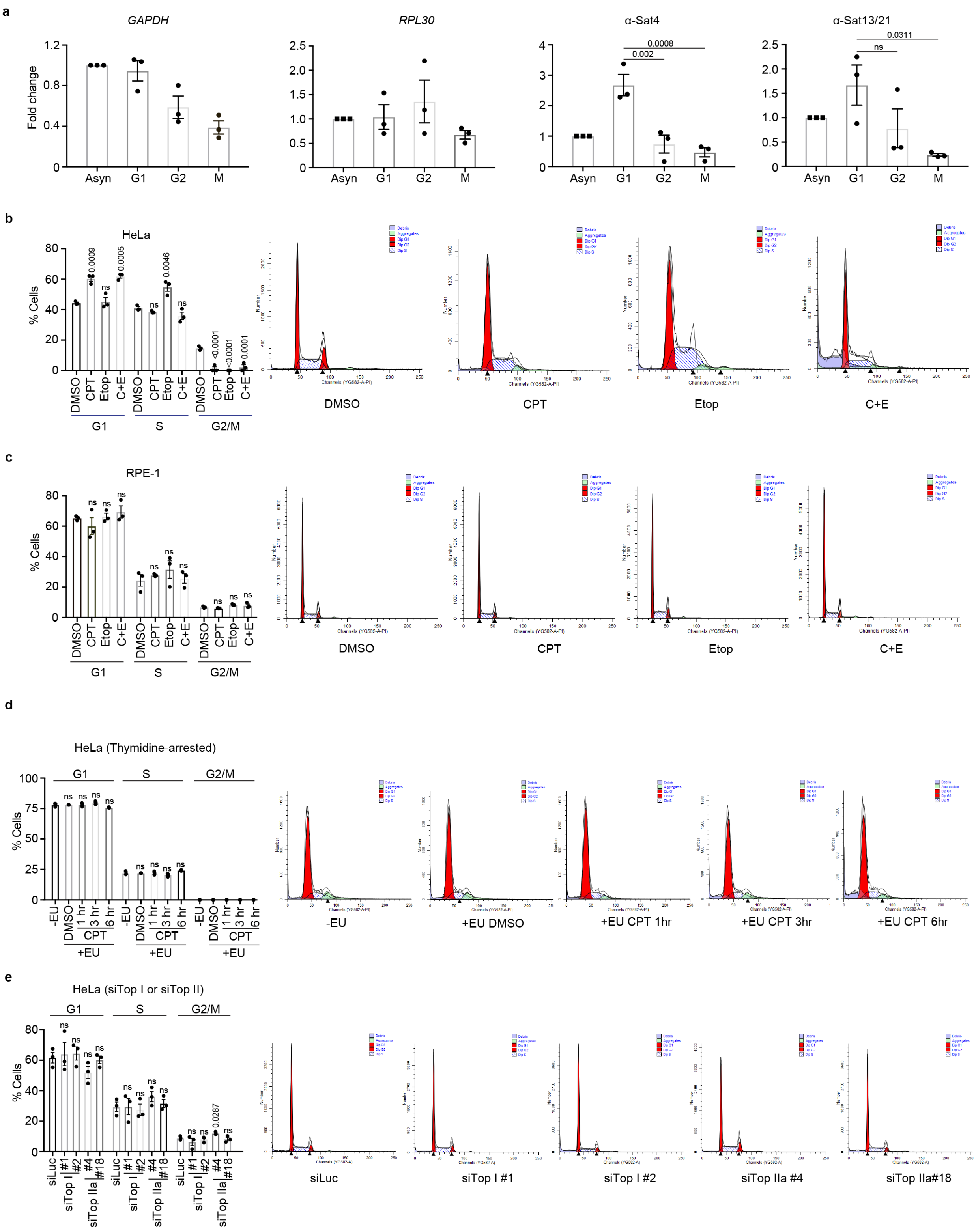

Supplementary Fig. 3. Flow-cytometric analyses of HeLa cells

a. α-satellite transcription is cell cycle-regulated. RNAs extracted for HeLa cells arrested with thymidine (18 hrs, G1), RO3306 (18 hrs, G2) or nocodazole (16 hrs, M), were subjected to real-time PCR analysis. Biological replicates n=3. Data are presented as mean values +/- SEM**.** ANOVA followed by pairwise comparisons with Turkey's test**. b** and **c**. **Flow-cytometric analyses of HeLa cells with chemical treatments.** HeLa (**b**) or RPE-1 (**c**) cells treated with DMSO, CPT (2.5 μM), Etoposide (Etop, 30 μM), or CPT plus Etoposide (C+E) for 12 hrs, were analyzed with FACS. Quantifications of cell cycle profiles were shown in the left panel. Biological replicates (n=3). Representatives of FACS results are shown in the right panels. Data are presented as mean values +/- SEM**.** ANOVA followed by pairwise comparisons with Turkey's test**. d. Flow-cytometric analyses of Thymidine-arrested HeLa cells with CPT treatment at different timepoints.** HeLa cells were incubated with thymidine for 18 hrs, during which, cells were also treated with EU and/or CPT (2.5 μM) at different timepoints. Cells were then collected for FACS analysis. Biological replicates n=3. Data are presented as mean values +/- SEM**.** ANOVA followed by pairwise comparisons with Turkey's test. **e. Flow-cytometric analyses of HeLa cells transfected with TopI or TopII siRNAs.** HeLa cells transfected with luciferase, TopI or TopII siRNAs for 48 hrs, were collected for FACS analysis. Biological replicates n=3. Data are presented as mean values +/- SEM**.** Two-sided Student's T-test. ns, not significant (P>0.1). Numeric values for P<0.1 are shown. The source data are provided in the Source Data file. The strategy for FACS analysis here is shown in Supplementary Fig. 11.

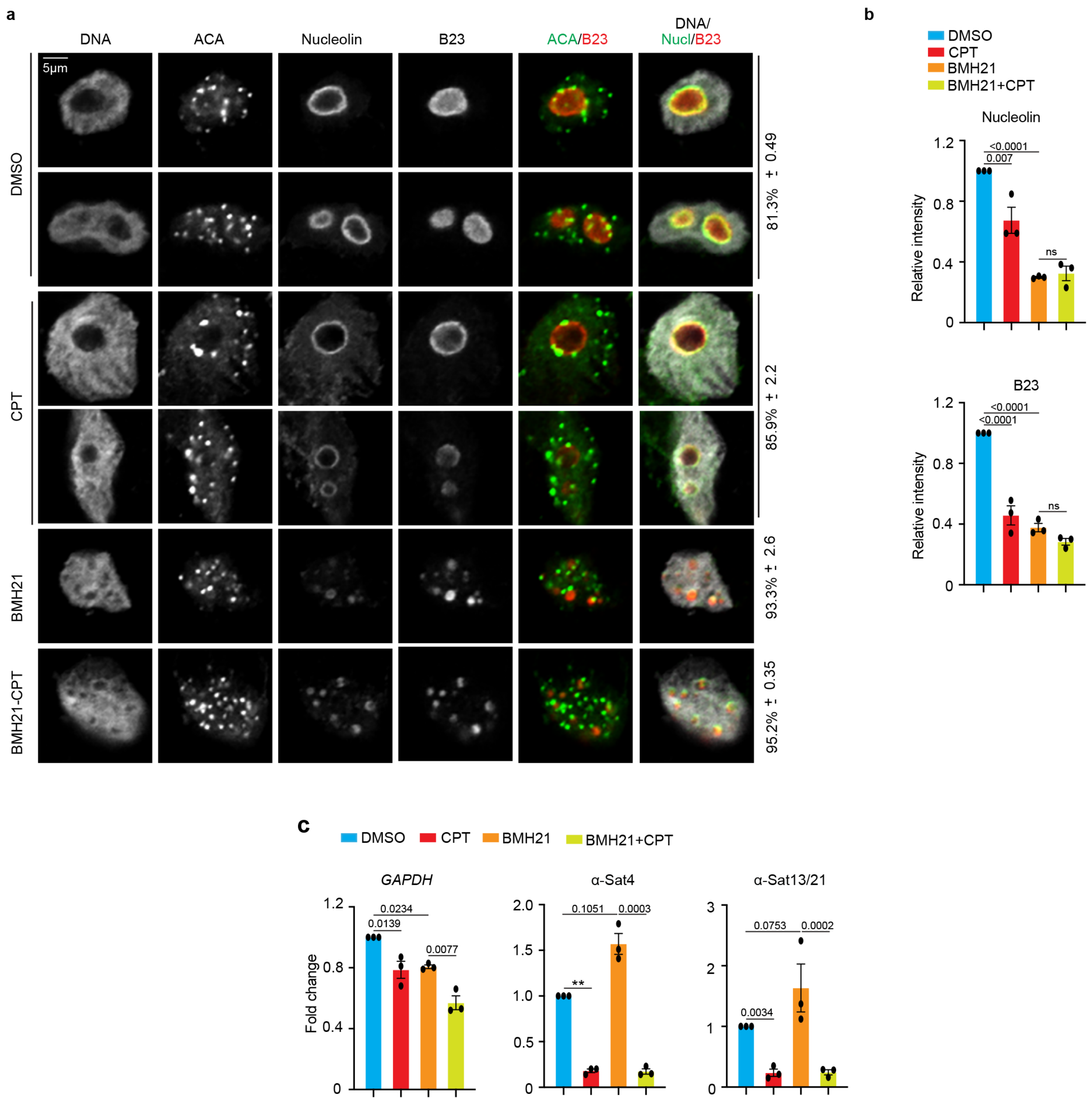

Supplementary Fig. 4. The nucleolus may not play an important role in TopI-mediated α-satellite transcription

a. RNAP I inhibitor BMH-21 treatment disrupts the nucleolar structure and decreases the levels of nucleolin and B23. HeLa cells treated with DMSO, CPT (10 μM), BMH-21 (RNAPI inhibitor), or BMH-21 plus CPT for 12 hr were stained with DAPI and the indicated antibodies. The percentages in the left panel represent cells with specific nucleolus morphology. Biological replicates n=3. Data are presented as mean values +/- SEM. Representative images are shown here. **b.** Relative intensities of nucleolar components nucleolin (nucleolin/DNA) and B23

(B23/DNA) in (**a**) are shown here. Biological replicates n=3. Data are presented as mean values +/- SEM. ANOVA followed by pairwise comparisons with Turkey's test**. c**. **TopI-regulated α-satellite transcription is independent of intact nucleolar structures.** HeLa cells treated as described in (**a**) were collected for real-time PCR analysis. Biological replicates n=3. Data are presented as mean values +/- SEM. ANOVA followed by pairwise comparisons with Turkey's test were applied. ns, not significant (P>0.1). Numeric values for P<0.1 are shown. The source data are provided in the Source Data file.

Primers	DMSO	Etop (Fold change)		
GAPDH	1.00	0.93		
RPL30	1.00	0.97		
D1Z5-b	1.00	2.81	Old	
D1Z5-c	1.00	10.74	Old	Centromere 1
D1Z7	1.00	71.12	Young	
D2Z1	1.00	206.58		
D3Z1	1.00	20.77		
D4Z1	1.00	162.11		
D5Z1	1.00	139.47		
D6Z1	1.00	196.75		
D7Z1	1.00	63.20	Young	Centromere 7
D7Z2	1.00	407.75	Old	
D8Z2	1.00	24.88		
D9Z4	1.00	27.50		
D10Z1	1.00	33.83		
D11Z1	1.00	261.85		
D12Z3	1.00	31.82		
D13Z1	1.00	1.17		
D13Z2	1.00	99.61		
D13Z3	1.00	6.11		
D13Z6	1.00	1.27		
D13Z7	1.00	18.18		
D13Z8	1.00	2.72		
D13Z9	1.00	8.28		
D14Z1	1.00	36.74		
D14Z2	1.00	55.56		
D14Z3	1.00	35.30		
D15Z3	1.00	22.18		
D16Z2	1.00	87.87		
D17Z1	1.00	128.83	Young	Centromere 17
D17Z1-b	1.00	29.20	Old	
D18Z1	1.00	30.23		
D18Z2	1.00	12.10		
D19Z4	1.00	1.71		
D19Z5	1.00	0.55		
D20Z2	1.00	51.70		
D21Z1	1.00	1.15		
D22Z4-1	1.00	95.65		
D22Z4-2	1.00	2.80		
D22Z5	1.00	10.83		
DXZ1	1.00	33.14		

Supplementary Fig. 5. Real-time PCR CT values for different kinds of α-satellite arrays and etoposide globally induces α-satellite transcription

Real-time PCR analyses of centromere RNAs using primers against distinct types of α-satellite HORs. RNAs extracted from etoposide-treated HeLa cells (30 μM, 12 hrs) were subjected to real-time PCR analysis using primers against different types of. α-satellite HORs. Two boxed regions in red are α-satellite HORs for centromeres 1 and 20. Young and old denote youngest α-satellite HOR and oldest α-satellite HOR, as described in[20].

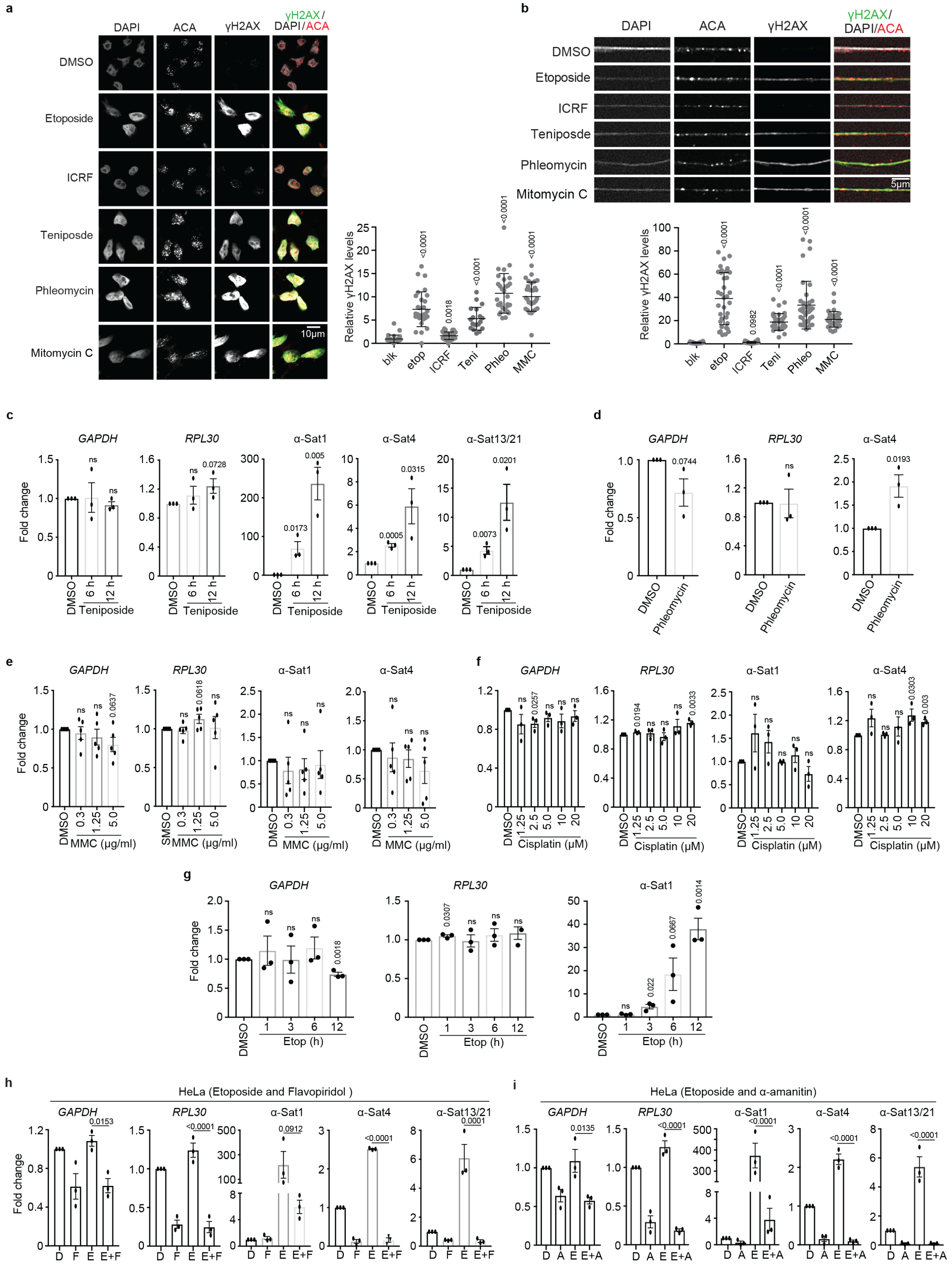

Supplementary Fig. 6. DSBs dramatically increases α-satellite transcription

a. DNA-damage analyses of HeLa cells with chemical treatments. HeLa cells were treated with DMSO, etoposide (Etop, 30 μM), ICRF (20 μM), teniposide (Teni, 2.5 μM), phleomycin (phleo, 80 μg/ml), or mitomycin C (MMC, 5 μg/ml) for 12 hrs, and then subjected to immunostaining. Quantitation of γH2AX levels (γH2AX/DNA) was shown in the right panel. Similar results were obtained in two biological replicates and the statistical analysis was carried out based on a single experiment . Each dot represent one cell. n=19 for Teni and n=30 for others. Data are presented as mean values +/- SD. Two-sided Student's T-test**. b. DNA-damage analyses of centromeres in HeLa cells with chemical treatments**. HeLa cells treated with DMSO, etoposide (Etop) (12 hrs, 30 μM), ICRF (20 μM), teniposide (Teni) (12 hrs, 2.5 μM), phleomycin (Phleo) (24 hrs, 80 μg/ml), or mitomycin C (MMC) (12 hrs, 5 μg/ml), were subjected to chromatin stretch followed by immunostaining. Quantitation or γH2AX levels (γH2AX/DNA) on centromeric regions was shown in the bottom panel. Similar results were obtained in two biological replicates and the statistical analysis was carried out based on a single experiment . Each dot represents one cell. n=35 for etop, n=34 for MMC and n=36 for others. Data are presented as mean values +/- SD. Two-sided Student's T-test. **c**, **d**, **e**, and **f, Effects of various chemicals on α-satellite transcription.** HeLa cells were treated with DMSO, teniposide (**c**) (12 hrs, 2.5 μM), phleomycin (**d**) (24 hrs, 80 μg/ml), mitomycin c (MMC, **e**) (12 hrs, indicated concentrations), or cisplatin (**f**) (12 hrs, indicated concentrations), and RNAs were extracted for real-time PCR analysis. Results for α-Sat13/21 primers are shown in Fig.3d. Biological replicates (n=5 for (**e**) and n=3 for others). Data are presented as mean values +/- SEM**.** Two-sided Student's T-test**. g. Etoposide increases α-satellite transcription in a time-dependent manner.** HeLa cells were treated with DMSO or etoposide (2.5 μM) at the different timepoints and RNAs were extracted for real-time PCR analysis. Results from α-Sat13/21 primers were recorded in Fig.3e. Biological replicates n=3. Data are presented as mean values +/- SEM**.** Two-sided Student's T-test**. h** and **i. DSB-induced α-satellite transcription depends on RNAP II.** HeLa cells treated with RNAP II inhibitor α-amanitin (**i,** 50 μg/ml), flavopiridol (**h,** 1 μM), etoposide (**h** and **i**, 30 μM), flavopiridol plus etoposide (**h**), or α-amanitin plus etoposide (**i**) for 12 hrs and RNAs were extracted for real-time PCR analysis. Biological replicates n=3. Data are presented as mean values +/- SEM. ANOVA followed by pairwise comparisons with Turkey's test. ns, not significant (P>0.1). Numeric values for P<0.1 are shown. The source data are provided in the Source Data file.

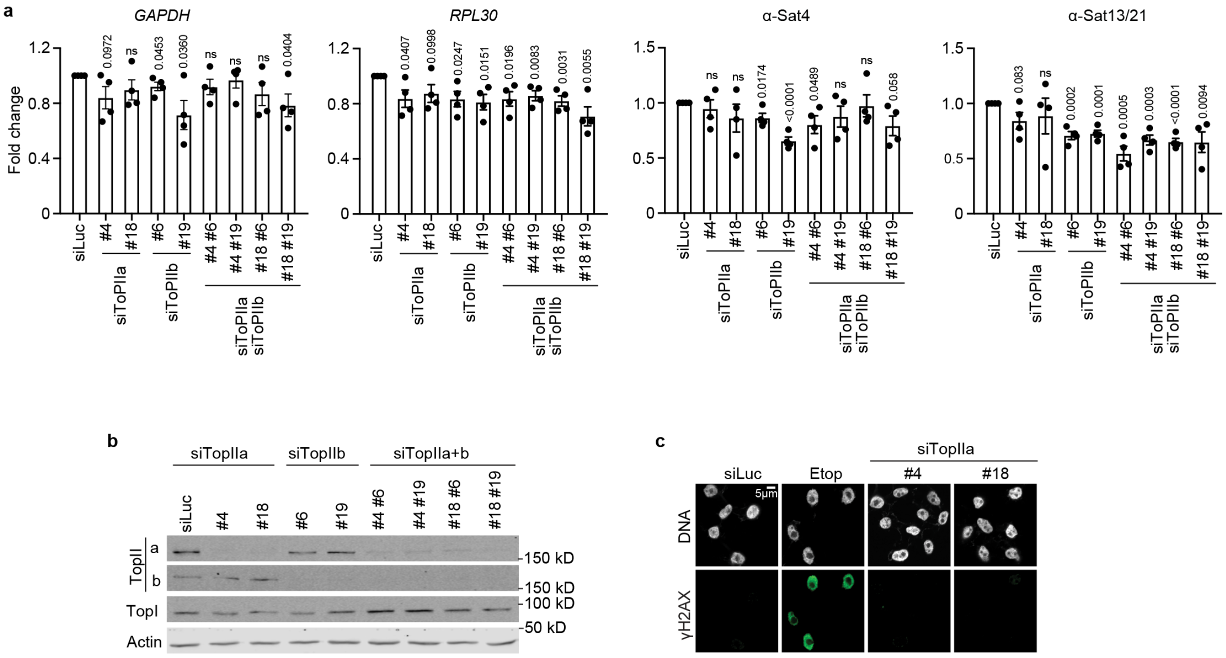

Supplementary Fig. 7. TopII knockdown by siRNAs does not affect α-satellite transcription

a. TopII knockdown barely changes α-satellite transcription. RNAs from HeLa cells transfected with the indicated siRNAs were subjected to real-time PCR analysis. The statistical analysis was carried out based on various independent biological replicates (n=4). Data are presented as mean values +/- SEM**.** Two-sided Student's T-test**. b. Protein levels of TopII in TopII knockdown cells.** Lysates of HeLa cells in (**a**) were subjected to Western-blotting analysis. **c. TopII knockdown does not significantly induce DNA damage**. HeLa cells were transfected with the indicated siRNAs and then subjected to immunostaining with the indicated antibodies. ns, not significant (P>0.1). Numeric values for P<0.1 are shown. The source data are provided in the Source Data file.

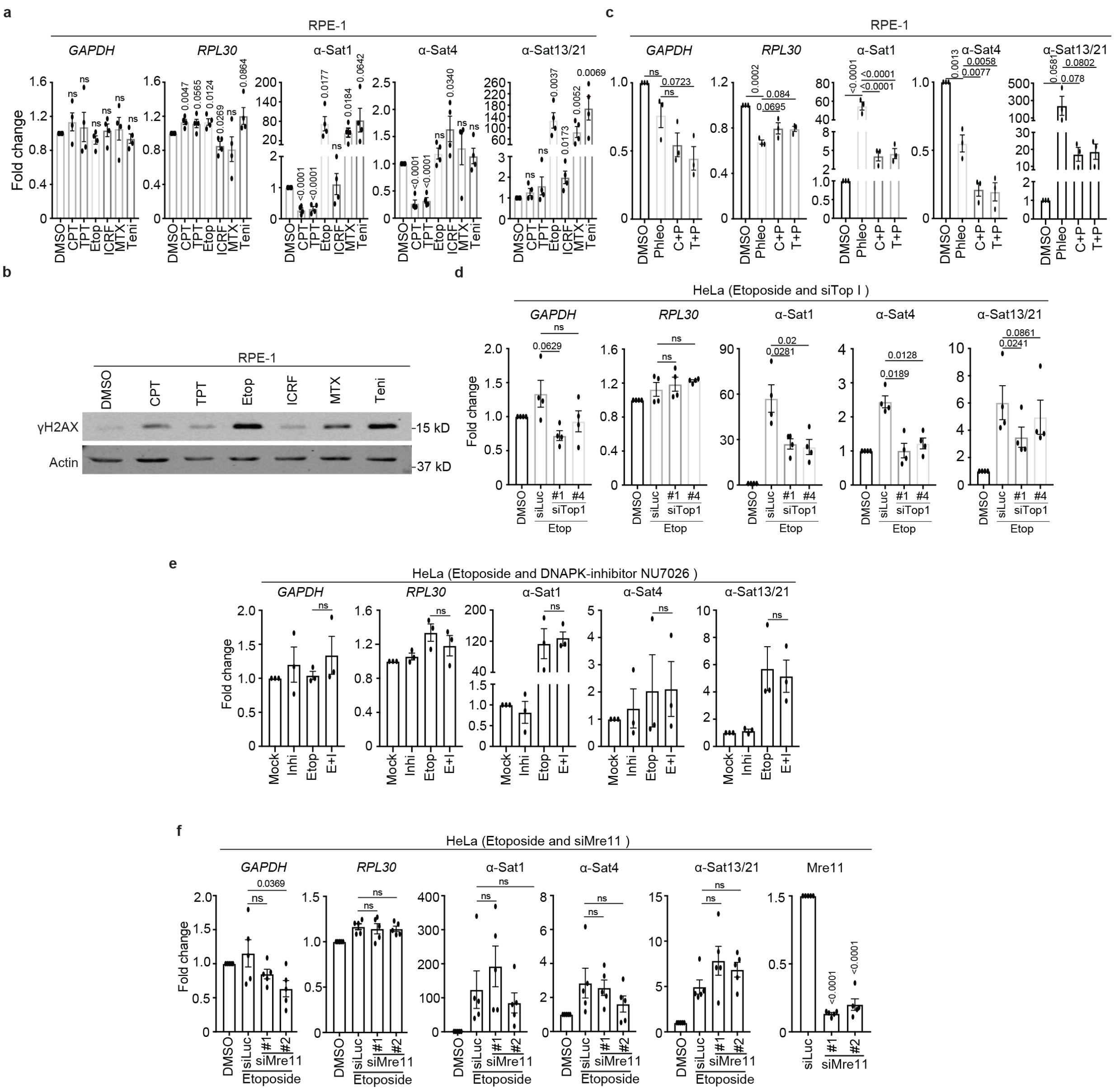

Supplementary Fig. 8. TopI regulates DSB-increased α-satellite transcription

a. DSB increases α-satellite transcription in RPE-1 cells. RNAs from RPE-1 cells treated with CPT (2.5 μM), TPT (10 μM), etoposide (Etop, 30 μM), ICRF (20 μM), methotrexate (MTX, 2.0 μM), or teniposide (Teni, 2.5 μM) for 12 hrs were subjected to real-time PCR analysis. The statistical analysis was carried out based on various independent biological replicates (n=4). Data are presented as mean values +/- SEM**.** Two-sided Student's T-test **. b. γH2AX levels in (a).** Lysates of RPE-1 cells in (**a**) were subjected to SDS-PAGE resolution and then blotted with the indicated antibodies. Similar results were observed in two biological replicates. **c. DSB-**

induced α-satellite transcription depends on TopI. RNAs from RPE-1 cells treated with DMSO, phleomycin (Phleo) (24 hrs, 80 μg/ml), CPT (12 hrs, 2.5 μM) plus phleomycin (C+P), or TPT (12 hrs, 10 μM) plus phleomycin (T+P) were subjected to real-time PCR analysis. The statistical analysis was carried out based on various independent biological replicates (n=3). Data are presented as mean values +/- SEM**.** ANOVA followed by pairwise comparisons with Turkey's test**. d. TopI knockdown by siRNAs partially decreases α-satellite transcription.** HeLa cells transfected with luciferase or TopI siRNAs were treated with etoposide (Etop, 30 μM) for 12 hrs and RNAs were extracted for real-time PCR analysis. The statistical analysis was carried out based on various independent biological repeats (n=4). Data are presented as mean values +/- SEM**.** Two-sided Student's T-test**. e. DNAPK is dispensable for DSB-induced α-satellite transcription.** RNAs extracted from HeLa cells treated with DMSO, DNA-PK inhibitor NU7206 (0.8 μM), etoposide (Etop, 30 μM), etoposide plus NU7206 (E+I) were subjected for real-time PCR analysis. The statistical analysis was carried out based on various independent biological replicates (n=3). Data are presented as mean values +/- SEM**.** ANOVA followed by pairwise comparisons with Turkey's test**. f. Mre11 is dispensable for DSB-induced α-satellite transcription by etoposide.** HeLa cells transfected with luciferase or Mre11 (#1 or #2) siRNAs were treated with etoposide (30 μM) for 12 hrs. RNAs were extracted for real-time PCR analysis. The statistical analysis was carried out based on various independent biological repeats (n=5). Data are presented as mean values +/- SEM**.** ANOVA followed by pairwise comparisons with Turkey's test. ns, not significant (P>0.1). Numeric values for P<0.1 are shown. The source data are provided in the Source Data file.

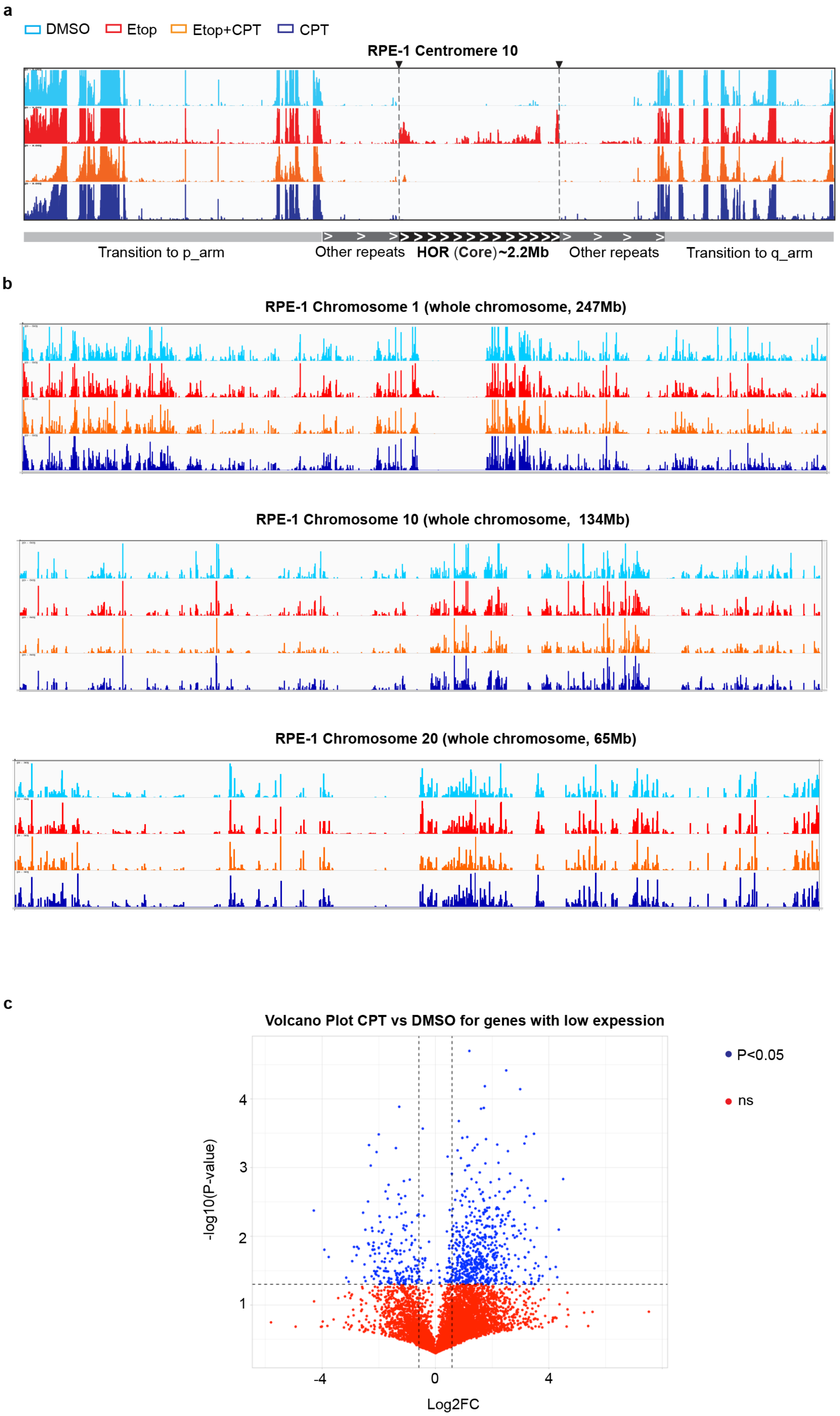
a
DMSO
Etop
Etop+CPT
CPT
RPE-1 Centromere 10
Transition to p_arm
Other repeats
HOR (Core)~2.2Mb
Other repeats
Transition to q_arm
b
RPE-1 Chromosome 1 (whole chromosome, 247Mb)
RPE-1 Chromosome 10 (whole chromosome, 134Mb)
RPE-1 Chromosome 20 (whole chromosome, 65Mb)
c
Volcano Plot CPT vs DMSO for genes with low expression
-log10(P-value)
Log2FC
-4
0
4
1
2
3
4
P<0.05
ns

Supplementary Fig. 9. RNA-seq analyses of HeLa cells with treatments of various chemicals

RPE-1 cells were treated with DMSO, CPT (2.5 μM), Etoposide (Etop, 30 μM), or etoposide plus CPT for 12 hrs and total RNAs were extracted for RNA-Seq analysis. Technical details were recorded in the section of Methods. **a. RNA-seq analysis of α-SatRNAs for centromere 10.** Results for centromeres 1 and 20 were recorded Fig. 4f. **b. CPT treatment does not significantly change gene expression profile.** RNA-seq analyses were performed for gene expression profile on chromosomes 1, 10 and 20. **c. Analysis of changes in the expression of lowly transcribed genes between CPT and DMSO treatments.** RNA-seq analyses were performed to examine the transcriptional change for lowly transcribed genes after CPT treatment. Details are recorded in the section of Methods.

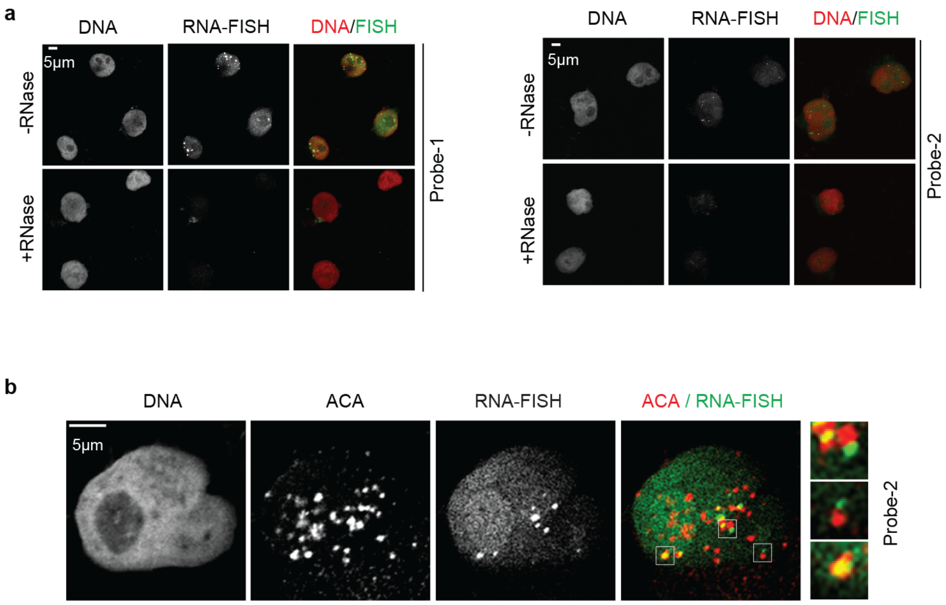

Supplementary Fig. 10. α-satRNA-FISH analyses of HeLa cells with various treatments

a. **α-satRNA-FISH fluorescence signals are sensitive to RNase treatment.** Etoposide-treated **(**30 μM, 12 hrs**)** HeLa cells were incubated with RNase before being subjected to hybridization with fluorescence-labelled probe-1 (left) and probe-2 (right). **b. DSB-induced α-satRNA-FISH foci do not always localize to centromeres.** Etoposide-treated **(**30 μM, 12 hrs**)** HeLa cells were hybridized with probe-2 and stained with ACA. Selected regions were amplified and placed in the right panel.

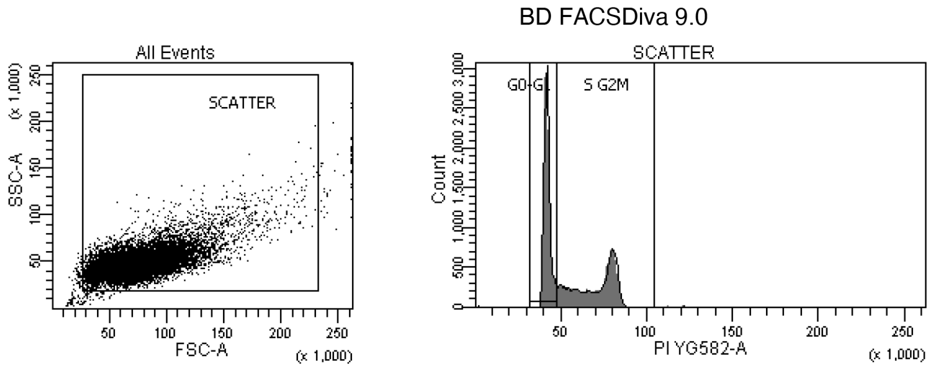

Experiment Name:	07282022 LY CELL CYCLE_Hela-RPE1
Specimen Name:	Hela-3rd
Tube Name:	MOCK
Record Date:	Jul 28, 2022 1:21:39 PM

Population	#Events	%Parent	PI YG582-A Median
G0-G1	12,061	49.2	40,459
S G2M	11,672	47.6	72,742

Tube: MOCK

Population	#Events	%Parent	%Total
All Events	25,000	####	100.0
SCATTER	24,504	98.0	98.0
G0-G1	12,061	49.2	48.2
S G2M	11,672	47.6	46.7

Supplementary Fig. 11. Gating strategy for FACS analysis

In FACS analysis in Supplementary Fig. 3, a gate was applied to include as many cells as possible. Further gates were applied to define G1 cells, S cells, G2 cells, cell debris, and cell aggregates. Distributions of these cell populations were revealed by color-shaded areas in each sample (supplementary Fig. 3). Percentages of G1 cells, S cells and G2 cells were calculated based on the color-shaded areas.
