# Supplementary material for: Topoisomerase I is an evolutionarily conserved key regulator for satellite DNA transcription
Source: Nat Commun. 2024 Jun 17;15:5151. doi: 10.1038/s41467-024-49567-5 (PMC11183047; doi:10.1038/s41467-024-49567-5)
Supplement: Supplementary file 3 — Reporting Summary [file 41467_2024_49567_MOESM3_ESM.pdf]

nature portfolio

Corresponding author(s): Hong Liu

Last updated by author(s): May 20,2024

Reporting Summary

Nature Portfolio wishes to improve the reproducibility of the work that we publish. This form provides structure for consistency and transparency in reporting. For further information on Nature Portfolio policies, see our Editorial Policies and the Editorial Policy Checklist.

Statistics

For all statistical analyses, confirm that the following items are present in the figure legend, table legend, main text, or Methods section.

n/a	Confirmed	
☐	☒	The exact sample size (*n*) for each experimental group/condition, given as a discrete number and unit of measurement
☐	☒	A statement on whether measurements were taken from distinct samples or whether the same sample was measured repeatedly
☐	☒	The statistical test(s) used AND whether they are one- or two-sided<br>*Only common tests should be described solely by name; describe more complex techniques in the Methods section.*
☒	☐	A description of all covariates tested
☒	☐	A description of any assumptions or corrections, such as tests of normality and adjustment for multiple comparisons
☐	☒	A full description of the statistical parameters including central tendency (e.g. means) or other basic estimates (e.g. regression coefficient) AND variation (e.g. standard deviation) or associated estimates of uncertainty (e.g. confidence intervals)
☐	☒	For null hypothesis testing, the test statistic (e.g. *F*, *t*, *r*) with confidence intervals, effect sizes, degrees of freedom and *P* value noted<br>*Give P values as exact values whenever suitable.*
☒	☐	For Bayesian analysis, information on the choice of priors and Markov chain Monte Carlo settings
☒	☐	For hierarchical and complex designs, identification of the appropriate level for tests and full reporting of outcomes
☒	☐	Estimates of effect sizes (e.g. Cohen's *d*, Pearson's *r*), indicating how they were calculated

Our web collection on statistics for biologists contains articles on many of the points above.

Software and code

Policy information about availability of computer code

Data collection	The images were taken by a Nikon inverted confocal microscope (Eclipse Ti2, NIS-Elements software). RNA-Seq data were collected using BGI DNBSEQ.
Data analysis	Image processing was carried out with ImageJ 1.53k and Adobe Photoshop 2022. Quantification was carried out with ImageJ. Statistical analysis was performed with Graphpad Prism 10.<br><br>Paired-end ribodepleted total RNA-seq reads were first validated by the FastQC algorithm. Raw sequence reads were then aligned to a complete Telomere-to-Telomere (T2T) reconstructed human reference genome (T2T-CHM13 v1.0) (Nurk et al., 2022). The alignments were performed using Spliced Transcripts Alignment to a Reference (STAR) aligner version 2.5.3a (Dobin et al., 2013) and were subjected to visual inspection using the Integrative Genomics Viewer (IGV) genome browser (Robinson et al., 2011). Transcript data from STAR were subsequently analyzed using RSEM version 1.3.0 (Li and Dewey, 2011) for quantification of human centromere transcripts. Read coverage data were generated using the bamCoverage tool and visualized using the IGV genome browser as previously described (Strong et al., 2014)

For manuscripts utilizing custom algorithms or software that are central to the research but not yet described in published literature, software must be made available to editors and reviewers. We strongly encourage code deposition in a community repository (e.g. GitHub). See the Nature Portfolio guidelines for submitting code & software for further information.

Data

Policy information about availability of data

All manuscripts must include a data availability statement. This statement should provide the following information, where applicable:

- Accession codes, unique identifiers, or web links for publicly available datasets
- A description of any restrictions on data availability
- For clinical datasets or third party data, please ensure that the statement adheres to our policy

All the relevant data are included in the main figures, Supplementary information and Source Data file. Original RNA-seq data have been deposited to NCBI SRA# SRP381962. There are not any restrictions on data availability. Source data are provided with this paper.

Research involving human participants, their data, or biological material

Policy information about studies with human participants or human data. See also policy information about sex, gender (identity/presentation), and sexual orientation and race, ethnicity and racism.

Reporting on sex and gender	N/A
Reporting on race, ethnicity, or other socially relevant groupings	N/A
Population characteristics	N/A
Recruitment	N/A
Ethics oversight	N/A

Note that full information on the approval of the study protocol must also be provided in the manuscript.

Field-specific reporting

Please select the one below that is the best fit for your research. If you are not sure, read the appropriate sections before making your selection.

☒ Life sciences ☐ Behavioural & social sciences ☐ Ecological, evolutionary & environmental sciences

For a reference copy of the document with all sections, see nature.com/documents/nr-reporting-summary-flat.pdf

Life sciences study design

All studies must disclose on these points even when the disclosure is negative.

Sample size	No specific statistical methods were used to estimate sample sizes. Three biological replicates were routinely used for most experiments, which is based on manufacturers' recommendations for use of commercially available reagents/assays or based on most previous publications in life science.
Data exclusions	No data was excluded from analysis
Replication	Experiments were usually performed at least three times unless specified. These are independent biological replicates unless specified.
Randomization	Cells grown in the different wells of a plate or in different petri dishes were randomly chosen for different treatments.
Blinding	Investigators were not blinded during data collection or analysis.

Reporting for specific materials, systems and methods

We require information from authors about some types of materials, experimental systems and methods used in many studies. Here, indicate whether each material, system or method listed is relevant to your study. If you are not sure if a list item applies to your research, read the appropriate section before selecting a response.

Materials & experimental systems

n/a	Involved in the study
☐	☒ Antibodies
☐	☒ Eukaryotic cell lines
☒	☐ Palaeontology and archaeology
☐	☒ Animals and other organisms
☒	☐ Clinical data
☒	☐ Dual use research of concern
☒	☐ Plants

Methods

n/a	Involved in the study
☒	☐ ChIP-seq
☐	☒ Flow cytometry
☒	☐ MRI-based neuroimaging

Antibodies

Antibodies used

Antibodies used in this study: anti-centromere antibody (ACA or CREST-ImmunoVision, HCT-0100), anti-Rpb1 (Abcam, ab5408), anti-Actin (Invitrogen, MA5-11869), anti-Rpb1-pSer2 (Biolegend, H5), anti-Rpb1-pSer2 (Active motif, 61083), anti-γH2AX (Cell signaling, 2577), anti-Top 1 (Bethyl, A302-589A), anti-Top IIa (Bethyl, A300-054A), anti-Top IIb (Bethl, A300-949A), anti-Top I-cc (MilliporeSigm MABE1084), anti-RNAP II (MilliporeSigm, 8WG16), anti-γH2Av (DHSB, UNC93-5.2.1)

Validation

Commercial antibodies are usually quality controlled by the manufacturer.
ACA or CREST-ImmunoVision, HCT-0100, https://calbiotech.com/products/human-antibody-against-centromere
anti-Rpb1 (Abcam, ab5408), https://www.abcam.com/products/primary-antibodies/rna-polymerase-ii-ctd-repeat-ysptsps-phospho-s5-antibody-4h8-chip-grade-ab5408.html
anti-Actin (Invitrogen, MA5-11869), https://www.thermofisher.com/antibody/product/Actin-Antibody-clone-ACTN05-C4-Monoclonal/MA5-11869
anti-Rpb1-pSer2 (Biolegend, H5), https://www.biolegend.com/en-us/products/purified-anti-rna-polymerase-ii-rpb1-antibody-13077
anti-Rpb1-pSer2 (Active motif, 61083), https://www.activemotif.com/catalog/details/61083/rna-pol-ii-ctd-phospho-ser2-antibody-mab
anti-γH2AX (Cell signaling, 2577), https://www.cellsignal.com/products/primary-antibodies/phospho-histone-h2a-x-ser139-antibody/2577
anti-Top IIa (Bethyl, A300-054A), https://www.fortislife.com/products/primary-antibodies/rabbit-anti-topo-ii-alpha-antibody/BETHYL-A300-054A
anti-Top IIb (Bethl, A300-949A), https://www.fortislife.com/products/primary-antibodies/rabbit-anti-topo-ii-beta-antibody/BETHYL-A300-949A
anti-Top I-cc (MilliporeSigm, MABE1084), https://www.emdmillipore.com/US/en/product/Anti-Topoisomerase-I-DNA-Covalent-Complexes-Antibody-clone-1.1A,MM_NF-MABE1084?ReferrerURL=https%3A%2F%2Fwww.google.com%2F
anti-RNAP II (MilliporeSigm, 8WG16), https://www.emdmillipore.com/US/en/product/Anti-RNA-Polymerase-II-Antibody-CTD-Antibody-clone-8WG16,MM_NF-05-952-I-100UG?ReferrerURL=https%3A%2F%2Fwww.google.com%2F
anti-γH2Av (DHSB, UNC93-5.2.1) , https://dshb.biology.uiowa.edu/UNC93-5-2-1

Eukaryotic cell lines

Policy information about cell lines and Sex and Gender in Research

Cell line source(s)

HeLa Tet-On (Invitrogen, human female); RPE-1 (ATCC, human male); NIH3T3 (ATCC, mouse male); S2 (Drosophila, Drosophila Genomics Resource Center)

Authentication

Cells were authenticated by the vendor when purchased. HeLa and RPE-1 were also authenticated using STR profiling in ATCC. NIH3T3 and S2 were gifts from other labs.

Mycoplasma contamination

HeLa and RPE-1 were tested negative for mycoplasma contamination . NIH3T3 and S2 were not tested for mycoplasma contamination.

Commonly misidentified lines
(See ICLAC register)

No commonly misidentified lines were used in this study.

Animals and other research organisms

Policy information about studies involving animals; ARRIVE guidelines recommended for reporting animal research, and Sex and Gender in Research

Laboratory animals	Drosophila melanogaster: w1118 strain w[1118] was used in this study (Bloomington stock center)
Wild animals	N/A
Reporting on sex	Both males and females
Field-collected samples	N/A
Ethics oversight	N/A

Note that full information on the approval of the study protocol must also be provided in the manuscript.

Plants

Seed stocks	N/A
Novel plant genotypes	N/A
Authentication	N/A

Flow Cytometry

Plots

Confirm that:

- [x] The axis labels state the marker and fluorochrome used (e.g. CD4-FITC).
- [x] The axis scales are clearly visible. Include numbers along axes only for bottom left plot of group (a 'group' is an analysis of identical markers).
- [x] All plots are contour plots with outliers or pseudocolor plots.
- [x] A numerical value for number of cells or percentage (with statistics) is provided.

Methodology

Sample preparation	Cultured cells that were harvested cells by trypsinization were washed with PBS (PH7.4), and fixed with ice-cold 70% ethanol overnight at −20°C. After ethanol was washed out with PBS, cells were further permeabilized with PBS (PH 7.4) containing 0.25% Triton X-100 for 5 min. Finally, cells were stained with propidium iodide (Sigma-Aldrich) at a final concentration of 20 μg/ml. RNase A (QIAGEN) was added at a final concentration of 200 μg/ml.
Instrument	BD LSR Fortessa flow cytometer
Software	Modfit
Cell population abundance	At least a few thousand cells for each sample were analyzed for fluorescence intensity.
Gating strategy	A gate was applied to include as many cells as possible (Supplementary Fig. 11). Further gates were applied to define G1 cells, S cells, G2 cells, cell debris, and cell aggregates (Supplementary Figs. 3 and 11). Distributions of these cell populations were revealed by color-shaded areas in each sample (see supplementary Fig. 3). Percentages of G1 cells, S cells and G2 cells were calculated based on the color-shaded areas.

- [x] Tick this box to confirm that a figure exemplifying the gating strategy is provided in the Supplementary Information.
